# Risk Factors and Clinical Presentation in Dogs with Increased Serum Pancreatic Lipase Concentrations—A Descriptive Analysis

**DOI:** 10.3390/ani12121581

**Published:** 2022-06-19

**Authors:** Harry Cridge, Nicole Scott, Jörg M. Steiner

**Affiliations:** 1 Department of Small Animal Clinical Sciences, Michigan State University College of Veterinary Medicine, East Lansing, MI 48824, USA; 2 Gastrointestinal Laboratory, Department of Small Animal Clinical Sciences, College of Veterinary Medicine and Biomedical Sciences, Texas A&M University, College Station, TX 77843, USA; nicolescott1320@tamu.edu (N.S.); jsteiner@cvm.tamu.edu (J.M.S.)

**Keywords:** canine, pancreatitis, cPLI, etiology, clinical signs

## Abstract

**Simple Summary:**

Over the past several years, there has been an increasing importance placed on clinical presentation as part of a diagnosis of pancreatitis, and yet there is comparatively little data investigating the full array of clinical signs that may be seen in dogs with pancreatitis. Our study showed that dogs with increased pancreatic lipase immunoreactivity concentrations could display a wide range of clinical signs, which may be related to pancreatitis or a concurrent disease. Non-specific clinical signs, such as anorexia, were prevalent. Additionally, overt abdominal pain was infrequently reported, and veterinarians should be cautious in ruling out pancreatitis due to a lack of abdominal pain alone. Additionally, limited data is available on potential risk factors for pancreatitis in dogs; this information could be important in the development of disease prevention strategies. In our study, the most common concurrent disease was hepatobiliary abnormalities. Additional studies are needed to determine whether this is a causative or associative relationship. Drug use reflected common prescribing practices, and anti-epileptic drug use was low despite prior studies documenting clear associations between phenobarbital and potassium bromide and drug-associated pancreatitis. Adult maintenance diets, in addition to human foods and dog treats, were commonly fed prior to the development of an increased pancreatic lipase concentration.

**Abstract:**

Limited data exist regarding the full array of clinical signs seen in dogs with pancreatitis and potential risk factors for the disease. Laboratory submissions from the Gastrointestinal Laboratory at Texas A&M University were retrospectively reviewed for dogs with an increased serum pancreatic lipase immunoreactivity (cPLI) concentration (≥400 µg/L), and an internet-based survey was distributed to the attending veterinarian and/or technician on each case. The survey contained questions related to (i) clinical signs, (ii) prior gastrointestinal upset, (iii) comorbidities, (iv) pre-existing medical therapies, and (v) dietary history. One hundred and seventy (170) survey responses were recorded. The top three clinical signs reported were inappetence (62%), diarrhea (53%), and vomiting (49%). Abdominal pain was noted in only 32% of dogs, likely associated with poor pain detection. Additionally, the majority of dogs (71%) had prior episodes of gastrointestinal upset within the past 12 months, lending support for the commonality of recurrent acute pancreatitis, or acute on chronic disease. Hepatobiliary abnormalities (24%) were the most common concurrent disease, and endocrine disorders were seen in a low proportion of respondents (5–8%). Adult maintenance diets (65%), dog treats (40%), and human foods (29%) were commonly consumed by dogs prior to the discovery of increased cPLI concentration.

## 1. Introduction

Pancreatitis is the most common disorder of the exocrine pancreas in dogs [1]. Histopathology is traditionally considered the gold standard diagnostic modality; however, it is not routinely used due to its invasive nature and inherent limitations, including the potential to miss localized lesions and challenges associated with determining the significance of subclinical histopathologic lesions [2,3]. Given this, there is a reliance on the use of a clinical gold standard, which comprises a combined assessment of history, clinical signs, diagnostic imaging, and serum pancreatic lipase immunoreactivity (cPLI) concentration. Many studies have investigated diagnostic imaging and cPLI assays; however, comparatively little data exists on clinical presentation. The largest study that focused on clinical signs of pancreatitis was published almost 25 years ago and utilized a histopathologic gold standard [4]. Other studies utilizing various diagnostic criteria and population sizes have reported varied frequencies of abdominal pain, vomiting, and diarrhea in pancreatitis [5,6,7]. Published data frequently represents referral-level populations. There is, therefore, a critical need to further investigate the range and frequency of clinical signs in dogs with increased serum cPLI concentrations across a multitude of practice types.

Additionally, pancreatitis in dogs is commonly considered idiopathic in nature, but several risk factors have been proposed [8]. Identification of risk factors would allow for the development of disease prevention strategies. Therefore, the aims of our study were to (i) document demographic data of dogs with an increased serum cPLI concentration, (ii) document the clinical presentation of dogs with an increased serum cPLI concentration, (iii) investigate the prevalence of comorbidities and pre-existing drug therapies in the study population, and (iv) document the dietary history of dogs with an increased serum cPLI concentration.

## 2. Materials and Methods

### 2.1. Case Identification and Survey Distribution

The submission records of a non-commercial laboratory (Gastrointestinal Laboratory, Texas A&M University, College Station, TX, USA) were retrospectively reviewed for dogs with an increased serum cPLI concentration (≥400 µg/L; measured by Spec cPL, Idexx Laboratories, Westbrook, ME, USA) between 1 November 2021, and 25 February 2022. This assay is not included in routine biochemical profiles and is specifically requested by the attending veterinarian on each case. Repeated measures were excluded. One thousand five hundred and thirty-one (1531) dogs were identified. Internet-based surveys (Qualtrics Version 16.0, Proto, UT, USA) were distributed via email, across four batches. Batching was used to ensure that surveys were distributed close to the time of sample collection to reduce recall bias. Surveys were completed by a licensed veterinarian or a licensed veterinary technician at the practice that submitted the serum sample. Multiple practice types were surveyed, including primary care clinics, emergency clinics, and specialty referral hospitals across the United States of America.

### 2.2. Data Handling and Analysis

Demographic data (i.e., breed, age, sex) were compiled from the laboratory records for all dogs with an increased serum cPLI concentration (≥400 µg/L) during the search period (*n* = 1531). Then data from the returned internet surveys (*n* = 170) were also reviewed under the following categories: (i) clinical signs, (ii) prior episodes of gastrointestinal upset, (iii) comorbidities, (iv) pre-existing medical therapies and nutraceuticals, and (v) dietary history. The survey utilized is available for review (Supplementary Material 1). Qualtrics (Qualtrics Version 16.0, Proto, UT, USA) was used for data compilation and calculation of percentages. Median and ranges were also calculated, and missing data were handled in accordance with the recommendations of an expert consultant on survey-based research. The denominator used for the calculation of percentages in question sub-analyses was based on the number of veterinarians that answered “yes” to at least one part of the question. This assumes that some veterinarians only entered “yes” rather than also utilizing the “no” and “unknown” columns, where available. Breed-related odds ratios (OR) were calculated using a two-sided Fisher’s exact test compared to a control population composed of the latest publicly available breed/litter registration data from the American Kennel Club (2009) [9]. Odds ratios were calculated against the total number of dogs from other breeds. Woolf logit interval was used to compute 95% confidence intervals (CI). Only significant odds ratios were reported. Only breeds with ≥ 5 dogs represented were included in the analysis. Analysis was performed using commercially available software (GraphPad Prism Version 9.0, GraphPad Software Inc., San Diego, CA, USA). Significance for all statistical comparisons was set at *p* < 0.05.

## 3. Results

### 3.1. Survey Population

A total of 1531 dogs were identified with a serum cPLI concentration (as measured by Spec cPL) ≥ 400 µg/L during the study period. Primary care practices represented 37% of samples, whereas 59% came from emergency or referral hospitals. Practice type could not be identified in 4% of samples. The median cPLI concentration was 763 µg/L (range, 400—≥ 2000 µg/L). Mixed breed dogs represented almost a third of dogs (451/1486, 30.3%). The most common breeds were Yorkshire Terriers (100/1486, 6.7%), Boxers (51/1486, 3.4%), and Labrador Retrievers (51/1486, 3.4%). Detailed demographic data are shown in Table 1. Body weight was unavailable for review.

The following breeds were at increased risk of an increased serum cPLI concentration: Greyhound (OR: 28.5, 95% CI: 13.92–58.47, *p* ≤ 0.001), Pointer (OR: 8.12, 95% CI: 3.84–17.17, *p* ≤ 0.001), Fox terrier (OR: 6.82, 95% CI: 3.65–12.77, *p* ≤ 0.001), Eskimo (OR: 6.67, 95% CI: 2.75–16.16, *p* = 0.001), Black and tan coonhound (OR: 3.40, 95% CI: 1.52–7.61, *p* = 0.01), Staffordshire terrier (OR: 2.70, 95% CI: 1.21–6.04, *p* = 0.03), Cardigan Welsh corgi (OR: 2.59, 95% CI: 1.08–6.26, *p* = 0.047), Cavalier King Charles spaniel (OR: 2.51, 95% CI: 1.85–3.40, *p* ≤ 0.001), Soft-coated wheaten terrier (OR: 2.48, 95% CI: 1.24–4.99, *p* = 0.02), Brussels griffon (OR: 2.44, 95% CI: 1.09–5.44, *p* = 0.04), and Miniature pinscher (OR: 2.33, 95% CI: 1.57–3.46, *p* ≤ 0.001) (See Table 2).

The following breeds were at decreased risk of an increased cPLI concentration: Beagle (OR: 0.19, 95% CI: 0.11–0.33, *p* ≤ 0.001), Labrador retriever (OR: 0.24, 95% CI: 0.18–0.32, *p* ≤ 0.001), Bulldog (OR: 0.26, 95% CI: 0.15–0.43, *p* ≤ 0.001), Golden retriever (OR: 0.32, 95% CI: 0.21–0.48, *p* ≤ 0.001), German shepherd dog (OR: 0.35, 95% CI: 0.25–0.50, *p* ≤ 0.001), Australian shepherd (OR: 0.35, 95% CI: 0.15–0.84, *p* = 0.01), Siberian husky (OR: 0.36, 95% CI: 0.17–0.75, *p* ≤ 0.001), German shorthair pointer (OR: 0.41, 95% CI: 0.22–0.77, *p* ≤ 0.001), Rottweiler (OR: 0.43, 95% CI: 0.25–0.74, *p* ≤ 0.001), Poodle (OR: 0.46, 95% CI: 0.29–0.71, *p* = ≤ 0.001), Pug (OR: 0.50, 95% CI: 0.28–0.88, *p* = 0.01), and Shih Tzu (OR: 0.64, 95% CI: 0.43–0.94, *p* = 0.02) (see Table 2). Non-significant odds ratios for breeds are not reported.

One-hundred and seventy (170) surveys were returned, indicating an 11.1% positive return rate. The results from the returned questionnaires are further described below.

### 3.2. Clinical Signs and Prior Episodes

The most common clinical sign reported was inappetence (92/148 responses, 62%), followed by diarrhea (78/148 responses, 53%) and vomiting (72/148 responses, 49%). Only approximately one-third of dogs had perceived abdominal pain or discomfort (48/148 responses, 32%). Complete data are displayed in Table 3.

Other stated clinical signs included weight loss (22 dogs), polydipsia and polyuria (11 dogs), neurologic compromise (5 dogs), respiratory compromise (3 dogs), borborygmus (5 dogs), and trembling (1 dog). The majority of dogs (105/148, 71%) had displayed prior episodes of gastrointestinal upset in the 12-month period immediately preceding serum sample collection. 

### 3.3. Comorbidities

The survey also enquired about the presence or absence of comorbidities as reported and diagnosed by the attending veterinarian. The three most common comorbidities were hepatobiliary abnormalities (35/146, 24%), kidney disease (27/146, 18%), and hypothyroidism (11/146, 8%). Complete data are displayed in Table 4.

Other stated comorbidities included chronic enteropathies (26 dogs), allergic skin disease (16 dogs), cardiac disease (12), urogenital disorders (11 dogs), seizures/other neurological disease (10 dogs), immune-mediated disease (5 dogs), cancer (5 dogs), orthopedic disease (5 dogs), hyperparathyroidism (2 dogs), systemic hypertension (2 dogs), and colorectal polyps (1 dog).

### 3.4. Pre-Existing Medical Therapies and Nutraceuticals

The most common pre-existing medical therapies received by dogs within 3 months of sample collection included supplements/nutraceuticals (51/143 responses, 36%), antibiotics (37/143 responses, 26%), and corticosteroids (24/143 responses, 17%). Complete data are displayed in Table 5. The most commonly stated nutraceuticals included joint supplements, followed by probiotics and anti-oxidants. The most common antibiotic reported to be administered was metronidazole. The most common anti-seizure medication reported to be administered was phenobarbital. The most common immunosuppressive agent, other than corticosteroids, reported to be administered was oclacitinib.

### 3.5. Dietary History (Prior to Sample Collection)

The top three nutrition sources fed to dogs prior to sample collection in the study were adult maintenance diets (80/123 responses, 65%), dog treats (49/123 responses, 40%), and human foods (36/123 responses, 29%). Of the dogs that received other therapeutic diets, renal/urinary diets were fed to 10 of the dogs. Complete data are displayed in Table 6. As dogs likely received more than one nutritional source, the predominant diet was also enquired about during the survey. The predominant diet type is shown in Table 7.

## 4. Discussion

In this descriptive survey-based study, we investigated the demographic data, clinical presentation, and potential risk factors for an increased serum pancreatic lipase immunoreactivity (cPLI) concentration ≥ 400 µg/L in a large cohort of dogs from multiple practice types. Increased serum cPLI concentration was utilized to determine study eligibility as it is an objective and highly specific biomarker of acute pancreatitis in dogs [10,11,12]. While other diagnostic criteria such as ultrasonographic features were initially considered in study planning, they were ultimately deemed more subjective than cPLI concentration, particularly in the face of a multi-practice evaluation. Ultrasound is well-known to be affected by operator experience, patient discomfort, and the information provided to the sonographer at the time of evaluation [13,14,15]. Clinical signs were not eligible for study inclusion due to the objectives of the study.

Mixed breed dogs represented almost one-third of dogs with an increased serum cPLI concentration. The top three affected breeds were Yorkshire terriers, Boxers, and Labrador retrievers. Mixed breed dogs and Labradors likely represent the common occurrence of this breed in the general dog population. Indeed, Labrador retrievers had a decreased risk of having an increased serum cPLI concentration relative to other breeds. Yorkshire terriers have also been identified as one of the most common breeds affected by pancreatitis in other studies, suggesting a breed-predisposition; however, a significant odds ratio was not detected in our study [4]. Boxers and Cavalier King Charles spaniels are reportedly predisposed to chronic pancreatitis, and this likely represents their over-representation in our study, particularly in the face of a high prevalence of prior clinical signs of gastrointestinal upset [16]. Cavalier King Charles spaniels had an almost 2.5× increased risk of having an increased serum cPLI concentration compared to other breeds. Other potentially predisposed breeds include the Black and tan coonhound, Brussels griffon, Cardigan Welsh corgi, Eskimo, Fox terrier, Greyhound, Miniature pinscher, Pointer, Soft-coated wheaten terrier, and Staffordshire terrier. Additional studies are needed on the causes of potential breed predispositions. No gender appeared to be over-represented in this study group, and most dogs were middle-aged to older, with a median age of 10.25 years, although a wide range of ages was reported.

The most common clinical sign reported in dogs with an increased serum Spec cPL concentration in this study was inappetence. This is important because it is often an underestimated clinical sign and has a broad list of differential diagnoses. Pancreatitis should therefore be considered an appropriate differential diagnosis for dogs with non-specific inappetence. The second most common clinical sign was diarrhea. The third most common sign was vomiting, but this was only seen in half of the study respondents. The prevalence of vomiting may also be related to the distribution of lesions within the pancreas [7]. Abdominal pain, which is commonly considered a cornerstone of acute pancreatitis, was only detected in around one-third of dogs in our study, which was a surprising finding given the prevalence (58–67%) reported in prior studies and anecdotal perception of dogs with pancreatitis [4,5,6]. Interestingly, these studies involved referral populations or fatal cases of pancreatitis, and the prevalence of abdominal pain has been reported to be as low as 15% in a study utilizing multiple practice types and a clinical diagnosis utilizing a point-of-care pancreatic lipase assay [7]. We therefore suspect that our data more accurately reflect the true prevalence of detected abdominal pain in dogs with increased serum cPLI concentrations. The percentage in this study likely reflects veterinarian and owner perception, rather than what is truly present. More studies are needed on pain perception in dogs with increased serum cPLI concentrations. It is the authors’ opinion that analgesics should be administered to all dogs with pancreatitis, even in the absence of overt abdominal pain, as abdominal discomfort is likely under-recognized. Other clinical signs commonly reported in the study population included lethargy, diarrhea, and nausea. Weight loss, polyuria/polydipsia, neurologic complications, respiratory compromise, borborygmi, and trembling were also intermittently reported. These clinical signs may represent a primary presentation of pancreatitis or may result from a comorbidity. The high proportion of animals with prior episodes of gastrointestinal upset suggests the presence of recurrent episodes of pancreatitis, acute on chronic disease, or pancreatitis occurring as a comorbidity to other intestinal disorders. Acute on chronic disease is a distinct potential given the high frequency of histopathologic evidence of chronic pancreatitis found in necropsy studies, which is otherwise undetected [16]. Long-term monitoring of cPLI concentrations and other biomarkers/imaging findings in dogs following an episode of pancreatitis is urgently required to further investigate this potential and its frequency.

Many diseases have been identified as potential risk factors for pancreatitis in dogs, including endocrinopathies, hypertriglyceridemia, infectious agents, and other miscellaneous disorders [8]. Hyperadrenocorticism (HAC) was reported in the history of 12/101 dogs with pancreatitis in one retrospective study, and in another study, dogs with HAC had higher cPLI concentrations than a control group of dogs [17,18]. Similar results have also been seen with other methodologies of pancreatic lipase quantification, e.g., 1-2-diglyceride and 1-2,o-dilauryl-rac-glycero-glutaric acid-(6′-methylresorufin) ester [19]. One study also documented a 4.5× increased risk of HAC in dogs with pancreatitis compared to those without pancreatitis [20]. In our study, a small, yet important, proportion of dogs with increased cPLI concentrations (≥400 µg/L) had a known history of hyperadrenocorticism. The exact pathophysiologic mechanism is not yet known [20]. Diabetes mellitus (DM) has also been reported in association with pancreatitis [20,21,22,23]. In our study, a small number of dogs with an increased cPLI concentration (≥400 µg/L) had a known history of diabetes mellitus. While the relationship between pancreatitis and DM is multidirectional, pancreatitis often leads to DM [24]. This is further supported by evidence of glucose intolerance in dogs with presumed chronic pancreatitis [25]. Hypertriglyceridemia may also mediate the relationship between DM and pancreatitis [20]. In our study, hypothyroidism was noted in a small number of dogs with an increased cPLI concentration (≥400 µg/L). Hypothyroidism has also been documented in 4/101 dogs in another retrospective study of pancreatitis in dogs [17]. Hyperlipidemia is thought to mediate any potential relationship between hypothyroidism and pancreatitis [22]. Hypothyroidism is also a common disease in middle-aged to older dogs, as with the signalment of dogs in this study. Other diseases reported in dogs with an increased serum cPLI concentration included hepatobiliary abnormalities, renal disease, hyperlipidemia, and tick-borne disease. The frequency of reported hepatobiliary abnormalities was somewhat unexpected but likely reflects reactive hepatic changes, post hepatic cholestasis, or less commonly functional cholestasis secondary to the cytokine response in pancreatitis, rather than pre-existing disease [26,27]. Reference criteria for the diagnosis of hepatic disease, including histopathology, were not provided. Thus, biochemical abnormalities alone may have been utilized to suggest the presence of hepatic disease. A similar caveat exists for renal disease. Hepatic lipase does not cross-react with the Spec cPL assay and is therefore unlikely to be the cause of the cPLI elevations in this study [28]. Timing of hepatic disease/biochemical abnormality relative to that of pancreatitis in each dog was unknown. Another study did, however, note a high prevalence of ultrasonographic evidence of pancreatitis (30.2%) in dogs with cholangitis or cholangiohepatitis [29]. Further research into this potential relationship is needed. The relationship between renal disease and pancreatitis is the source of much debate. Given the molecular weight of pancreatic lipase and a lack of a consistent relationship between pancreatic lipase and creatinine concentration, it is suspected that changes in GFR alone are not responsible for increased Spec cPL concentrations in dogs with renal disease [26,30,31]. Another study investigating DGGR lipase also noted a high prevalence of hyperlipasemia in dogs with naturally occurring renal disease [32]. Additional research is therefore needed to further investigate the relationship between naturally occurring renal disease and pancreatitis. Renal and urinary diets are often higher in dietary fat than standard adult maintenance diets, and this could be a contributing factor in this relationship; indeed, 10 dogs in our study were receiving such diets [33]. However, dietary fat level was not definitively assessed, and as such, this remains speculative. The renal disease reported may also represent acute kidney injury secondary to acute pancreatitis [34]. The low frequency of tick-borne disease likely reflects an incidental association. The risk factors studied in this project were determined based on a temporal association with the onset of pancreatitis. Given the data type, it is important to consider that these risk factors may not always represent a causative relationship and may instead be incidental or represent shared risk factors for disease. Additionally, the direction of a relationship cannot always be definitively determined.

Drug-associated pancreatitis (DAP) is an important etiology in humans with pancreatitis, and the Badalov classification system is used to identify potential at-risk drugs [35]. A recent review article highlighted the current dearth of knowledge on this topic in dogs and called for further reports of potential DAP [26]. Our data provides significant additional information regarding drug histories in dogs with increased serum cPLI concentrations. The top three most common types of medications used over a 3-month period prior to sample collection were antibiotics, corticosteroids, and non-steroidal anti-inflammatory drugs or related pharmaceuticals, e.g., grapiprant. Supplements and nutraceutics were also prescribed to over one-third of the study population. These are anecdotally among the most commonly prescribed drug classes, and as such, this finding may represent an incidental association. The most common antibiotic reported to have been administered was metronidazole, which is commonly used in the management of diarrhea (as seen in a large proportion of this study population), although this is associated with controversy and is no longer routinely recommended due to negative effects on the intestinal microbiome [36,37]. Corticosteroids have previously been implicated in the development of pancreatitis; however, the role of exogenous steroids in the etiology of pancreatitis is perpetually evolving [26]. Administration of corticosteroids to healthy dogs does not result in significant changes in cPLI concentrations or does not commonly (1/6) increase serum cPLI concentrations into a diagnostic range for pancreatitis [38,39]. Indeed, corticosteroids are now being used with increasing frequency in the management of acute pancreatitis [40,41]. In contrast with those results, our study showed a moderate frequency of corticosteroid use prior to sampling in dogs with increased Spec cPL concentrations, and this may reflect the presence of concurrent disease treated with corticosteroids or secondary pancreatic inflammation influenced by concurrent disease [8]. We cannot, however, rule out the potential that corticosteroids caused pancreatitis in some of the dogs in our study. Other drugs frequently implicated in the development of pancreatitis, such as phenobarbital and potassium bromide, were infrequently reported in our study population. This likely is related to a low frequency of anti-epileptic drug use in our population. Future studies should investigate drug latency periods and re-exposure data to provide further definitive evidence of DAP in dogs and to help develop a DAP classification scheme for use in veterinary patients. Latency is defined as the interval between starting a medication and pancreatitis induction, with a consistent latency period providing supportive evidence of DAP.

High-fat diets have long been suggested as a risk factor for pancreatitis in dogs based on early studies showing that high-fat diets induce or worsen the severity of pancreatitis in dogs [42,43]. However, limited data exist on naturally occurring disease, and a recent review concluded that there was insufficient evidence to make a definitive conclusion on the effects of high levels of dietary fat on the development of pancreatitis [8]. Adult maintenance diets were fed to the majority of respondents in the current study; however, dog treats and human foods were also fed to a significant proportion of the test population. Other diets commonly utilized in the management of gastrointestinal disorders were fed at a lower frequency, including therapeutic (not labeled low fat) gastrointestinal diets, hydrolyzed diets, and limited-ingredient novel protein diets. Diets labeled as low fat were fed to approximately one-fifth of the test population and may reflect the high frequency of prior gastrointestinal signs in the test population and subsequent prescribing practices of veterinarians. It is important to note that the level of dietary fat was not definitively assessed in this study. In a prior study of healthy dogs, there was no difference in Spec cPL concentrations between those dogs fed a maintenance diet (4.01 g of fat/100 kcal) and one labeled as a low-fat diet (1.55 g of fat/100 kcal) [44]. The high proportion of human foods and dog treats reflects anecdote and the results from a prior study, in which access to unusual food items, table scraps, and trash increased the risk of pancreatitis in dogs [45]. As this was a survey study, labeled claims as assessed by a licensed veterinarian or veterinary nurse/technician were utilized to determine which diets were low fat. The details required to calculate dietary fat on an energy basis were unavailable.

Additional limitations of our study are predominantly related to the nature of survey-based research. This study is an association study, and thus the level of evidence for a causative relationship should be considered weak. A control population and regression analysis would be required to determine true risk factors for pancreatitis. Although the survey was addressed to the veterinarian who submitted the sample and their nursing team, we cannot exclude the potential that someone else within the practice completed the survey based on the medical record. This could be a potential source of bias. Additionally, recall bias may have been introduced between the time of serum sampling and survey completion. The survey completion rate was 11.1%, and as such, data were not available from the majority of dogs with an increased serum cPLI concentration during the study period. Potential reasons for failure to complete the survey may include time constraints, lack of documented data in the medical record, or other factors. Additionally, dogs with milder clinical presentations (i.e., subclinical or mild disease) may not have had serum samples submitted to the laboratory for quantification of cPLI. Thus, as with other research, our study may have selected for more severe clinical presentations.

## 5. Conclusions

Dogs with increased pancreatic lipase immunoreactivity concentrations display a wide array of clinical signs. Of note, abdominal pain was infrequently reported and was likely under-detected. Pancreatitis should not be excluded based on a lack of perceived abdominal pain. Recurrent episodes of gastrointestinal upset are common in dogs with increased cPLI concentrations. Additional research on potential risk factors of pancreatitis, including potential DAP, is warranted.

## Figures and Tables

**Table 1:** Demographic details on the survey population. Note: Age was unavailable for 65 dogs. Sex status was unavailable for 31 dogs. Breed was unavailable for 45 dogs. Rounding resulted in a total > 100% for gender.

Variable	Dogs with cPLI ≥ 400 µg/L
Age (years), median (range)	10.25 (0.25–19)
Sex	79/1500 (5.3%) male intact 669/1500 (44.6%) male neutered 31/1500 (2.1%) female intact 721/1500 (48.1%) female spayed
Breed	Mixed breed (451) Yorkshire terrier (100) Boxer (51) Labrador retriever (51) Dachshund (46) Cavalier King Charles spaniel (43) Chihuahua (41) German shepherd (34) Miniature pinscher (30) Pomeranian (28) Miniature poodle (27) Cocker spaniel (26) Shih Tzu (26) Maltese (25) Miniature schnauzer (25) Golden retriever (23) French bulldog (20) Jack Russel terrier (20) Standard poodle (20) Other breeds with less than 20 dogs represented: Boston terrier (18), Great dane (16), Australian shepherd (15), Bulldog (14), Havanese (14), Beagle (13), Rottweiler (13), Shetland sheepdog (13), Border collie (12), Pug (12), Bichon frise (11), Papillon (11), Fox terrier (10), German shorthaired pointer (10), Greyhound (8), Soft-coated wheaten terrier (8), Basset hound (7), Bernese mountain dog (7), Pointer (7), Siberian husky (7), Bull terrier (6), Cairn terrier (6), Black and tan coonhound (6), Brussels griffon (6), Staffordshire terrier (Pit Bull) (6), West highland white terrier (6), Australian heeler (5), Belgian malinois (5), Eskimo (5), Lhasa apso (5), Toy poodle (5), Silky terrier (5), American staffordshire terrier (5), Vizsla (5), Cardigan eelsh corgi (5), Chesapeake bay retriever (4), Foxhound (4), Newfoundland (4), Shiba inu (4), Whippet (4), Catahoula leopard dog (3), English springer spaniel (3), Great pyrenees (3), Irish setter (3), Italian greyhound (3), Portuguese water dog (3), Border terrier (2), Boykin spaniel (2), Chinese crested (2), Collie (2), Doberman pinscher (2), English cocker spaniel (2), Keeshond (2), Manchester terrier (2), Pekingese (2), Redbone coonhound (2), Rhodesian ridgeback (2), Samoyed (2), Schipperke (2), Scottish terrier (2), Swiss mountain dog (2), Weimaraner (2), Pembroke welsh corgi (2), Affenpinscher (1), Airedale terrier (1), Akita (1), Alaskan malamute (1), Australian kelpie (1), Basenji (1), Belgian tervuren (1), Blue tick coonhound (1), Bouvier des flandres (1), Brittany spaniel (1), English setter (1), Mastiff (1), Norwegian elkhound (1), Norwich terrier (1), Plott hound (1), Curly-coated retriever (1), Saluki (1), Shar-pei (1), Tibetan terrier (1), and Welsh terrier (1).

**Table 2:** Breeds with a statistically significant increase or decrease in risk for an increased serum cPLI concentration.

Risk	Breed [Odds Ratio]
Increased Risk	Greyhound (OR: 28.5) Pointer (OR: 8.12) Fox terrier (OR: 6.82) Eskimo (OR: 6.67) Black and tan coonhound (OR: 3.40) Staffordshire terrier (OR: 2.70) Cardigan Welsh corgi (OR: 2.59) Cavalier King Charles spaniel (OR: 2.51) Soft-coated wheaten terrier (OR: 2.48) Brussels griffon (OR: 2.44) Miniature pinscher (OR: 2.33)
Decreased Risk	Beagle (OR: 0.19) Labrador retriever (OR: 0.24) Bulldog (OR: 0.26) Golden retriever (OR: 0.32) German shepherd dog (OR: 0.35) Australian shepherd (OR: 0.35) Siberian husky (OR: 0.36) German shorthair pointer (OR: 0.41) Rottweiler (OR: 0.43) Poodle (OR: 0.46) Pug (OR: 0.50) Shih Tzu (OR: 0.64)

**Table 3:** Clinical signs reported in returned survey data from dogs with a serum cPLI ≥ 400 µg/L.

Clinical Sign	No. of Dogs Affected
Inappetence	92/148 (62%)
Diarrhea	78/148 (53%)
Vomiting	77/148 (49%)
Lethargy	67/148 (45%)
Nausea	52/148 (35%)
Abdominal pain or discomfort	48/148 (32%)
Regurgitation	15/148 (10%)
Other clinical signs	55/148 (37%)

**Table 4:** Comorbidities reported in returned survey data from dogs with a serum cPLI concentration ≥ 400 µg/L.

Disease	No. of Dogs Affected
Hepatobiliary abnormalities (non-specified)	35/146 (24%)
Kidney disease (non-specified)	27/146 (18%)
Hypothyroidism	11/146 (8%)
Hyperadrenocorticism	8/146 (5%)
Diabetes mellitus	7/146 (5%)
Hyperlipidemia	6/146 (4%)
Tick-borne infections	3/146 (2%)
Foreign body (within 3 months)	1/146 (1%)
Other medical or surgical disorders	91/146 (62%)

**Table 5:** Pre-existing medical therapies and nutraceuticals reported in survey data from dogs with a serum cPLI concentration ≥ 400 µg/L.

Therapeutics	No. of Dogs
Supplements/Nutraceuticals	51/143 (36%)
Antibiotics	37/143 (26%)
Corticosteroids	24/143 (17%)
Non-steroidal anti-inflammatories (NSAIDs) or NSAID-like drugs	18/143 (13%)
Immunosuppressives (other than corticosteroids)	12/143 (8%)
Anti-seizure medications	3/143 (2%)
Chemotherapy drugs	1/143 (1%)

**Table 6:** Pre-existing diets reported in survey data from dogs with a serum cPLI concentration ≥ 400 µg/L. Note: some dogs did receive more than one diet type.

Dietary Type	No. of Dogs
Adult maintenance diet (over-the-counter)	80/123 (65%)
Commercial dog treats	49/123 (40%)
Human foods (table foods)	36/123 (29%)
Low-fat diet (based on label description)	23/123 (19%)
Other therapeutic diet(s)	18/123 (15%)
Gastrointestinal therapeutic diet (non-low fat)	18/123 (15%)
Homemade diet	16/123 (13%)
Hydrolyzed diet	9/123 (7%)
Limited-ingredient novel protein diet	8/123 (7%)

**Table 7:** Predominant diet type reported in survey data from dogs with a serum cPLI concentration ≥ 400 µg/L.

Predominant Diet Type	No. of Dogs
Adult maintenance diet (over-the-counter)	53/131 (40%)
Low-fat diet (based on label description)	22/131 (17%)
Gastrointestinal therapeutic diet (non-low fat)	15/131 (11%)
Other therapeutic diets	14/131 (11%)
Homemade diet	11/131 (8%)
Hydrolyzed diet	10/131 (8%)
Human foods (table foods)	3/131 (2%)
Commercial dog treats	2/131 (2%)
Limited-ingredient novel protein diet	1/131 (1%)

## Data Availability

Data are only available on request due to restrictions, e.g., privacy or ethical reasons. As the study identified animals with an increased serum cPLI concentration, no owner consent was signed, and personal data are therefore not publicly available.

## Supplementary Materials

The following supporting information can be downloaded at: https://www.mdpi.com/article/10.3390/ani12121581/s1, Supplementary Material 1.
