# Supplementary material for: Risk Factors and Clinical Presentation in Dogs with Increased Serum Pancreatic Lipase Concentrations—A Descriptive Analysis
Source: Animals (Basel). 2022 Jun 19;12(12):1581. doi: 10.3390/ani12121581 (PMC9219463; doi:10.3390/ani12121581)
Supplement: Supplementary file 1 [file animals-12-01581-s001.zip › animals-1757885-supplementary.pdf]

animals

MDPI

Supplementary Material 1: Distributed Survey Questions (*Adapted for offline display)

1) Participation in this study is voluntary and you will not be penalized if you elect not to participate. Anonymized and collated data from this project may be published. Do you provide informed consent for this survey?

    a. Yes

    b. No

2) I am a licensed veterinarian or veterinary nurse/technician

    a. Yes

    b. No

3) Which of the following clinical signs were noted at the time of presentation/sample collection?

	Yes	No	Unknown
Vomiting	○	○	○
Regurgitation	○	○	○
Nausea (e.g. lip licking, drooling)	○	○	○
Inappetence	○	○	○
Lethargy	○	○	○
Diarrhea	○	○	○
Abdominal pain/discomfort	○	○	○

4) Were any other clinical signs noted?

    a. Yes

    b. No

5) (If yes to Q4 was noted) Please briefly describe the additional clinical signs reported in the dog?

____

____

6) In the past 12 months has the dog displayed any other prior episodes of gastrointestinal upset (e.g., vomiting, diarrhea, nausea, etc.)?

    a. Yes

    b. No

7) Does the dog have any of the following comorbidities?

	Yes	No
Cushing's disease/ Hyperadenocorticism	○	○
Hypothyroidism	○	○
Diabetes mellitus	○	○
Hyperlipidemia/Hypertriglyceridemia	○	○
Liver disease	○	○
Kidney disease	○	○
Tick-borne infections	○	○
Gastrointestinal foreign body within past 3 months	○	○

8) Has the dog been diagnosed with any other medical or surgical diseases during his or her lifetime?

   a. Yes

   b. No

9) (If yes to Q8 was noted) Please list the other medical or surgical diseases that the dog has been diagnosed with. Include an approximate timeline if possible.

____

____

10) **Prior to the onset of clinical signs,** was the dog receiving any of the following medications (past 3 months)?

	Yes	No
Antibiotics	○	○
Anti-seizure medications	○	○
Chemotherapy drugs	○	○
Corticosteroids (steroids)	○	○
Immunosuppressives (other than steroids)	○	○
Non-steroidal anti-inflammatory drugs (or related)	○	○
Supplements or nutraceuticals	○	○

11) (If antibiotics were selected in Q10). Which antibiotics has the dog received in the past 3 months? Please list dose (mg/kg), dose frequency, and route of administration if possible.

____

____

12) (If anti-seizure medications were selected in Q10). Which anti-seizure medication has the dog received in the past 3 months? Please list dose (mg/kg), dose frequency, and route of administration if possible.

____

____

13) (If chemotherapy was selected in Q10). Which chemotherapy drug has the dog received in the past 3 months? Please list dose (mg/kg), dose frequency, and route of administration if possible.

____

____

14) (If corticosteroid/steroid was selected in Q10). Which corticosteroid/steroid has the dog received in the past 3 months? Please list dose (mg/kg), dose frequency, and route of administration if possible.

____

____

15) (If non-corticosteroid immunosuppressive agent was selected in Q10). Which immunosuppressive agent (other than steroids) has the dog received in the past 3 months? Please list dose (mg/kg), dose frequency, and route of administration if possible.

____

____

16) (If NSAID or related drug was selected in Q10). Which non-steroidal anti-inflammatory or related drug has the dog received in the past 3 months? Please list dose (mg/kg), dose frequency, and route of administration if possible.

____

____

17) (If supplements or nutraceuticals was selected in Q10). Which supplements or nutraceutical drugs has the dog received in the past 3 months?

____

____

18) Which of the following food types was eaten by your patient **<u>prior to the onset of clinical signs?</u>**

	Yes	No
Adult maintenance diet	○	○
"Low fat" diet	○	○
Routine "gastrointestinal" diet (not low fat)	○	○
Hydrolyzed diet	○	○
Novel protein diet	○	○
Home made diet	○	○
Human foods	○	○
Dog treats	○	○
Other prescription diet	○	○

19) (If adult maintenance diet was selected in Q18). Please state the adult maintenance diet fed to this patient

____

____

20) (If "low fat" diet was selected in Q18). Please state the "low fat" diet fed to this patient

____

____

21) (If routine gastrointestinal (non-low fat) diet was selected in Q18). Please state the "gastrointestinal" (non-low fat) diet fed to this patient

____

____

22) (If hydrolyzed diet was selected in Q18). Please state the hydrolyzed diet fed to this patient

____

____

23) (If novel protein diet was selected in Q18). Please state the novel protein diet fed to this patient

____

____

24) (If homemade diet was selected in Q18). Was the homemade diet formulated by a veterinary nutritionist?

    a. Yes – via a nutrition referral or in-house boarded nutritionist
    b. Yes – via a pre-published recipe
    c. No
    d. Unknown

25) (If human foods was selected in Q18). Which of the following human foods are fed to the dog?

	Yes	No
Meat	○	○
Vegetables/grains	○	○
Fruit	○	○
Dairy products	○	○
Eggs	○	○
Snacks	○	○
Bread	○	○
Other food items	○	○

26) (If other foods was selected in Q18). Which other food items are fed to the dog?

____

27) (If other prescription diet was selected in Q18). Please state the other type of prescription diet.

28) What is the predominant diet fed to the dog?

	Adult maintenance diet	Low fat diet	GI diet	Hydrolyzed	Novel Protein	Home made diet	Human foods	Dog treats	Other prescription diet
Which is the predominant diet?	○	○	○	○	○	○	○	○	○
